# Supplementary material for: Defect-Rich Heterogeneous MoS2/rGO/NiS Nanocomposite for Efficient pH-Universal Hydrogen Evolution
Source: Nanomaterials (Basel). 2021 Mar 8;11(3):662. doi: 10.3390/nano11030662 (PMC8001468; doi:10.3390/nano11030662)
Supplement: Supplementary file 1 [file nanomaterials-11-00662-s001.pdf]

nanomaterials

MDPI

Supplementary Materials

# Defect-Rich Heterogeneous $MoS_2$/rGO/NiS Nanocomposite for Efficient pH-Universal Hydrogen Evolution

**Guangsheng Liu, Kunyapat Thummavichai, Xuefeng Lv, Wenting Chen, Tingjun Lin, Shipeng Tan, Minli Zeng, Yu Chen, Nannan Wang * and Yanqiu Zhu**

Guangxi Institute Fullerene Technology (GIFT), Guangxi Key Laboratory of Processing for Non-Ferrous Metals and Featured Materials, Ministry of Education, School of Resources, Environment and Materials, Guangxi University, Nanning 530004, China; Jesson_lgs@163.com (G.L.); oluwafunmilola.ola1@nottingham.ac.uk (K.T.); Betsy_Lv@163.com (X.L.); wt15578102973@126.com (W.C.); w15260260610@163.com (T.L.); 1739200127@st.gxu.edu.cn (S.T.); MinliZeng_97@163.com (M.Z.); yc465@exeter.ac.uk (Y.C.); Y.zhu@exeter.ac.uk (Y.Z.)

* Correspondence: Wangnannan@gxu.edu.cn

## Table of Contents

**1. Experimental Procedures**

**2. Supplementary Figures**

**3. Supplementary Tables**

**4. Reference**

## 1. Experimental Procedures

### *1.1. Materials:*

All the chemicals were analytical reagents and used without further purification. L-Cysteine monohydrochloride (L-ch) and sodium molybdate dihydrate ($Na_2MoO_4·2H_2O$) were obtained from Sinopharm Chemical Reagent Co., Ltd. (Shanghai, China) Nickel sulfate hexahydrate ($NiSO_4·6H_2O$), potassium hydroxide (KOH) and sodium hydroxide (NaOH) were purchased from Guangdong Guanghua Sci-Tech Co., Ltd in Guangdong China. Sulfuric acid ($H_2SO_4$) was from Enox Co., Ltd. (Suzhou, China) Pt/C (20 wt% Pt) were purchased from Suzhou Yilongsheng Energy Technology Co., Ltd (Suzhou, China). Nafion perfluorinated resin (5 wt%) solution was purchased from Sigma-Aldrich (Missouri, USA).

### *1.2. Synthesis of αNi(OH)₂ Nanowires*

The $\alpha Ni(OH)_2$ nanowires was synthesized by the hydrothermal method [1]. Briefly, 20 mmol of $NiSO_4·6H_2O$ and 20 mmol of NaOH were dissolved in deionized water (40 mL) and stirred vigorously for 30 min to obtain homogeneous solution. Then, the NaOH solution was slowly poured into the $NiSO_4·6H_2O$ aqueous solution with stirring for 5 min. Well-mixed solution was transferred into a 100 mL Teflon-lined stainless steel autoclave and kept at 120 °C for 24 h (heat rate 3 °C $min^{-1}$). After naturally cooled at room temperature, the as-prepared $\alpha Ni(OH)_2$ was collected by centrifugation and washed several times with distilled water. The obtained products were dispersed in 200 mL deionized water to form a homogeneous suspension of nickel hydroxide with concentration of 4mg $mL^{-1}$.

### *1.3. Synthesis of $MoS_2$/rGO/NiS Nanocomposite*

For the synthesis of $MoS_2$/rGO/NiS catalyst, 5 mL $\alpha Ni(OH)_2$ suspension was added into 60 mL of ethanol/water (V:V = 1:1) solution. After stirring for 5 min, 2 mL GO solution was dispersed in the $\alpha Ni(OH)_2$ solution and treated with ultrasonication for 30 min. Subsequently, 281 mg of L-ch and 78 mg $Na_2MoO_4·2H_2O$ were added into the $\alpha Ni(OH)_2$/GO solution and kept at stirring for 30 minutes. The final solution was then transferred into a 100 mL Teflon-lined stainless autoclave. After reactions at 200 °C for 24h, the black precipitates were centrifuged and washed with ethanol and deionized water several times, then dried at 80 °C for 12h. The sample is labeled as $MoS_2$/rGO/NiS-X (X=0, 3, 5, 7) according to the volume of $\alpha Ni(OH)_2$ added (0, 3, 5 and 7 wt%). Pristine $MoS_2$ is prepared with L-ch and $Na_2MoO_4·2H_2O$ with a molar ratio (L-ch:$Na_2MoO_4$ = 2:1) under the same hydrothermal conditions.

### *1.4. Characterization*

Morphologies and microstructures of the as-prepared samples were analyzed using field emission scanning electron microscope (SEM, SU8020, Tokyo, Japan) operating at a voltage of 20 kV, and high-resolution transmission electron microscope (HR-TEM, Tecnai G2 F30, Oregan, USA) at 300 kV. Energy disperse X-ray (EDS) mapping attached to TEM was conducted to estimate the elemental composition and distribution of catalysts. The elemental microanalysis and atom binding states were examined by X-ray photoelectron spectroscope (XPS, ESCALAB 250XI, Waltham, USA) with an Al K$\alpha$ radiator. X-ray diffraction (XRD, SMARTLAB, Tokyo, Japan) pattern of catalysts were recorded on an X-ray diffractometer with a Cu K$\alpha$ radiation ($\lambda$ = 1.5406 Å). Nitrogen sorption isotherms was carried out using an Automatic specific surface area analyzer at −196 °C (TriStar II 3020, Georgia, USA). All samples were outgassed at 150 °C for 3h in a dynamic vacuum before measurement. The Brunauer Emmett Teller (BET) specific surface area was calculated from adsorption data. Raman spectra of as-obtained sample were examined with a 6 mW laser power at a 532 nm laser excitation (inVia Reflex, London, UK).

### *1.5. Electrochemical Measurements*

The HER performance of catalyst was performed via an electrochemical workstation (CHI 660E, ChenHua Instruments Co. Ltd., Shanghai, China) in a three-electrode system. Saturated calomel electrode (in 0.5 M $H_2SO_4$ and 1 M PBS) and Hg/HgO (1 M KOH) electrode were used as reference electrode. A graphite rob was used as a counter electrode for all electrochemical experiments. A glassy carbon electrode/rotating disc electrode (RDE) loaded with various catalysts was used as a working electrode at a rotation speed of 1600 rpm during the experiment. The homogeneous catalyst ink was prepared by dispersing 5 mg catalysts in 1 mL of mixture solvent containing 5 μL Nafion solution, 780 μL DI-water and 200 μL ethanol followed by ultrasonication for 30 min. Then 5 μL catalyst ink was loaded onto a GC electrode (mass loading~0.353 mg $cm^{-2}$) and dried naturally at an ambient temperature. Before the electrochemical measurement, the electrolyte solution was bubbled with nitrogen for at least 30 min to remove dissolved oxygen. Linear sweep voltammetry (LSV) was performed in $H_2SO_4$ (pH = 0.4), KOH (pH = 14) and PBS (pH = 7.4) solution with a scan sweep rate of 5 mV $S^{-1}$ from 0 to −0.6 V vs. RHE. Tafel plot was acquired by fitting the linear portion of the polarization curve. Cyclic voltammetry (CV) measurements for the stability of the catalysts was performed by taking 1000 CV cycles at a scan rate of 100 mV $S^{-1}$ (potential windows in $H_2SO_4$ and PBS solution are −0.4–0.2V vs SCE and in KOH solution is −0.4–0.1V vs Hg/HgO), and then LSV was measured again under same initial conditions. Electrochemical impedance spectra (EIS) was performed at various overpotentials with frequency from 0.1 to 100,000 Hz at the amplitude voltage of 5 mV. To evaluate the electrochemical active surface area of the catalysts, the double-layer capacitances ($C_{dl}$) was measured by CV with scan rates of 40, 80, 120, 160, 200 mV $S^{-1}$ (potential windows in acidic, alkaline and neutral media are 0.36–0.46V vs RHE, 0.6–0.7V vs RHE and 0.7–0.8V, respectively). All potentials data were calibrated to a reversible hydrogen electrode (RHE): E(RHE)=E(SCE) + $E^{\theta}$ + 0.059PH V ($E^{\theta}$ = 0.244 in 0.5 M $H_2SO_4$ and 1 M PBS solution, $E^{\theta}$ = 0.098 in 1 M KOH). All the ohmic potential drop caused by solution resistance has been corrected with 95% iR-correction.

## 2. Supplementary Figures

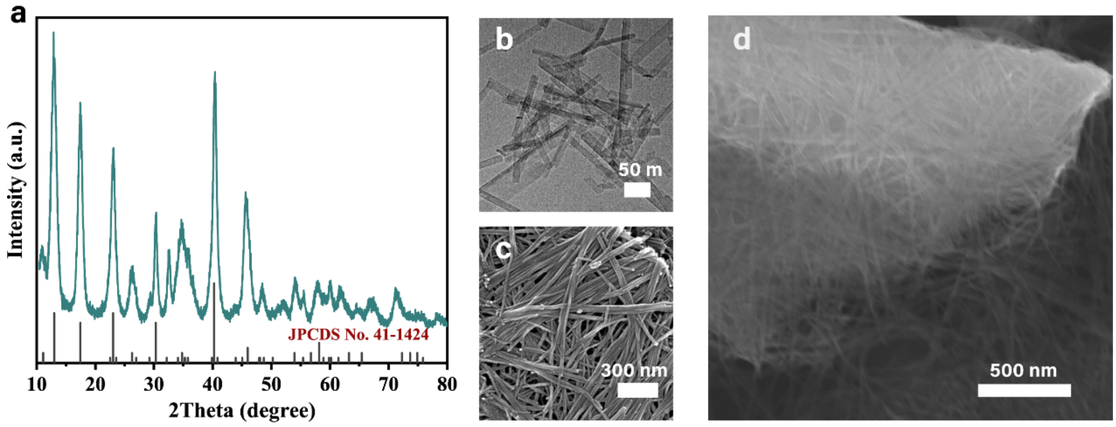


**Figure S1.** (**a**) XRD spectrum (**b**) TEM images and (**c**) SEM images of αNi(OH)$_2$ nanowires (**d**) SEM image of αNi(OH)$_2$ adsorbed on GO.

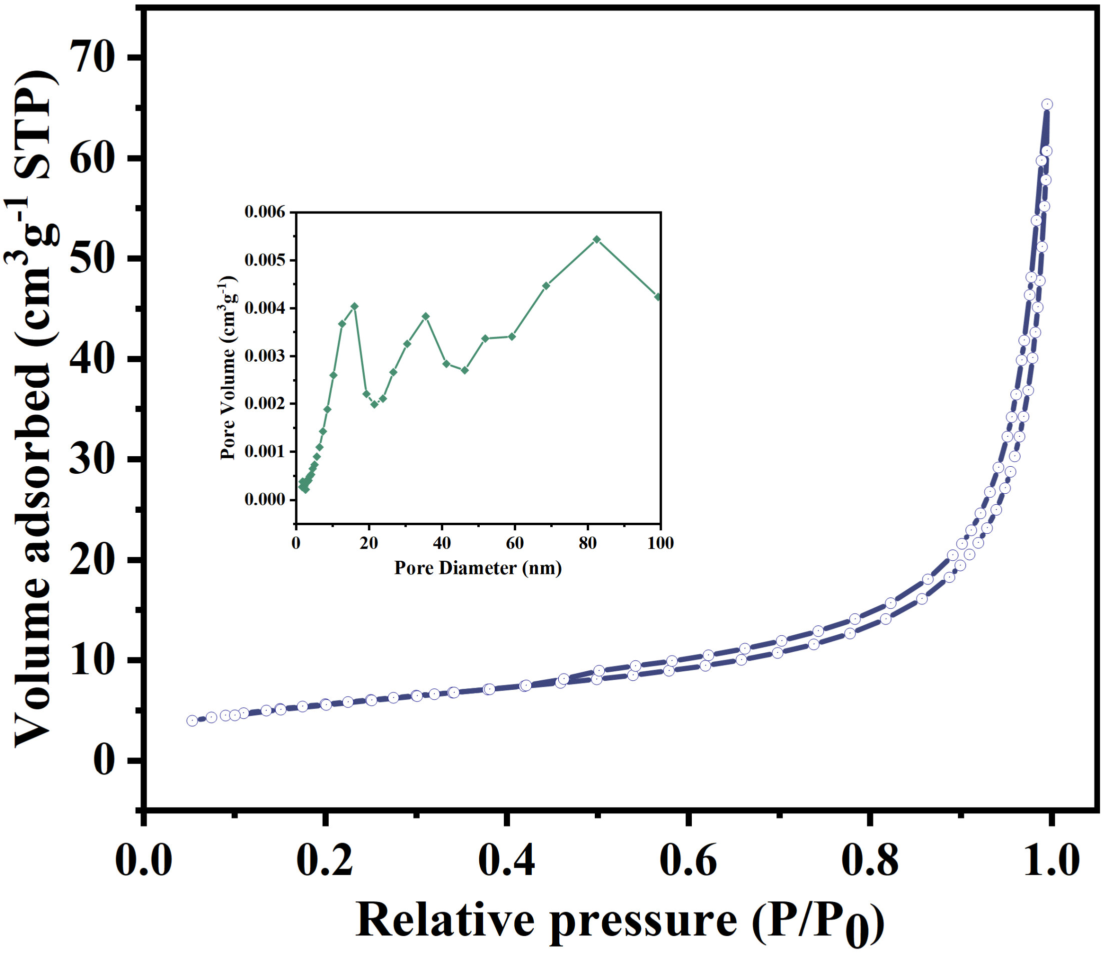


**Figure S2.** $N_2$ adsorption-desorption isotherms and the corresponding pore size-distribution profiles of $MoS_2$/rGO/NiS-5.

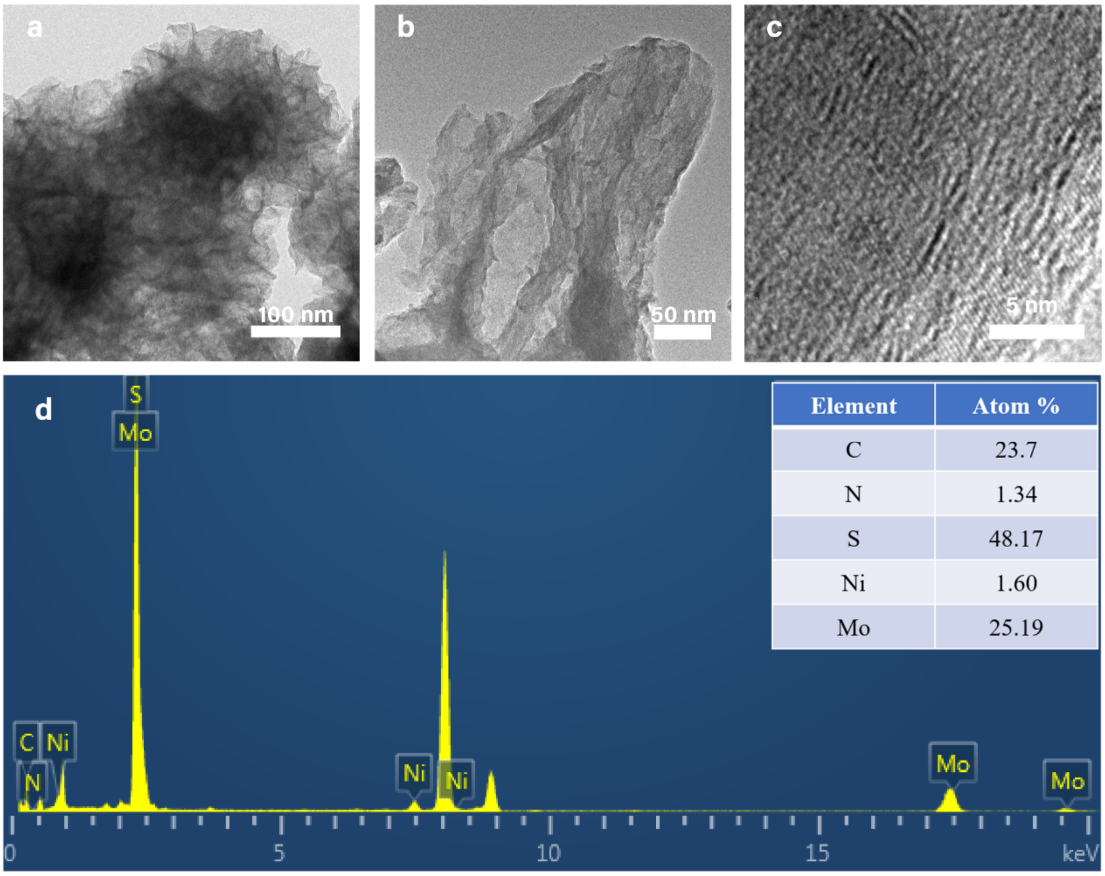


**Figure S3.** (**a**–**c**) Low magnification TEM images and (**d**) TEM-EDS pattern of $MoS_2$/rGO/Ni-5.

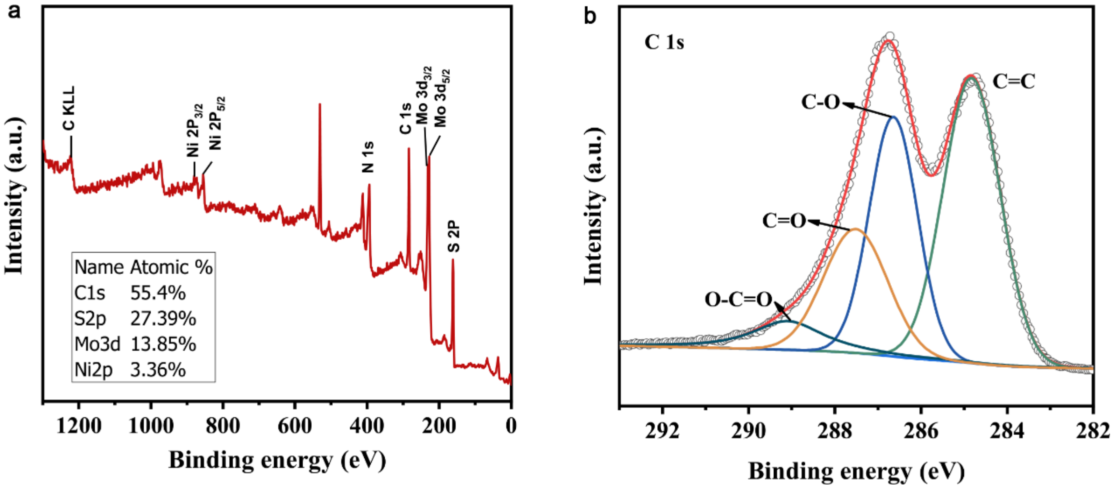


**Figure S4.** **(a)** The survey XPS spectrum of $MoS_2$/rGO/NiS-5; (**b**) XPS spectrum of C 1s of GO.

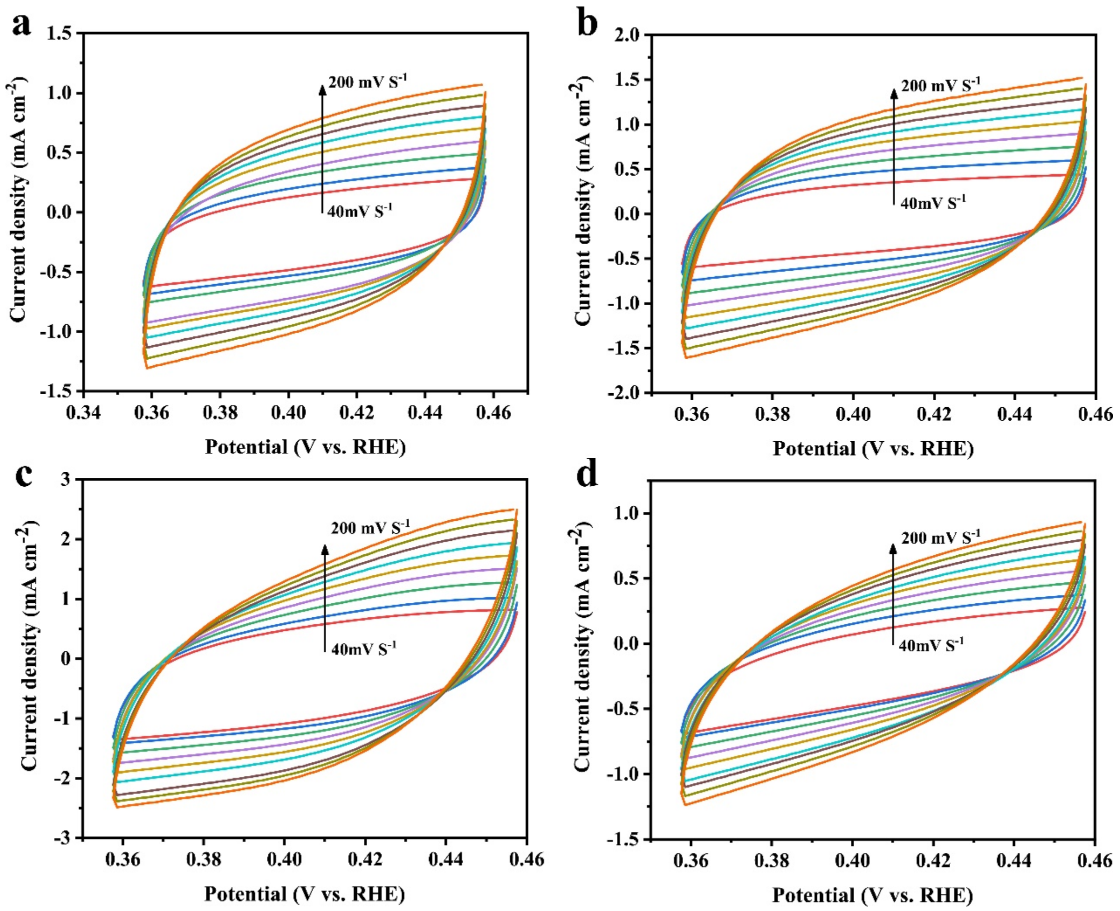


**Figure S5.** (**a**–**d**) Cyclic voltammograms curves of the $MoS_2$/rGO/NiS-X (X=0, 3, 5, 7) in 0.5 M $H_2SO_4$ under 0.36–0.46 V vs. RHE.

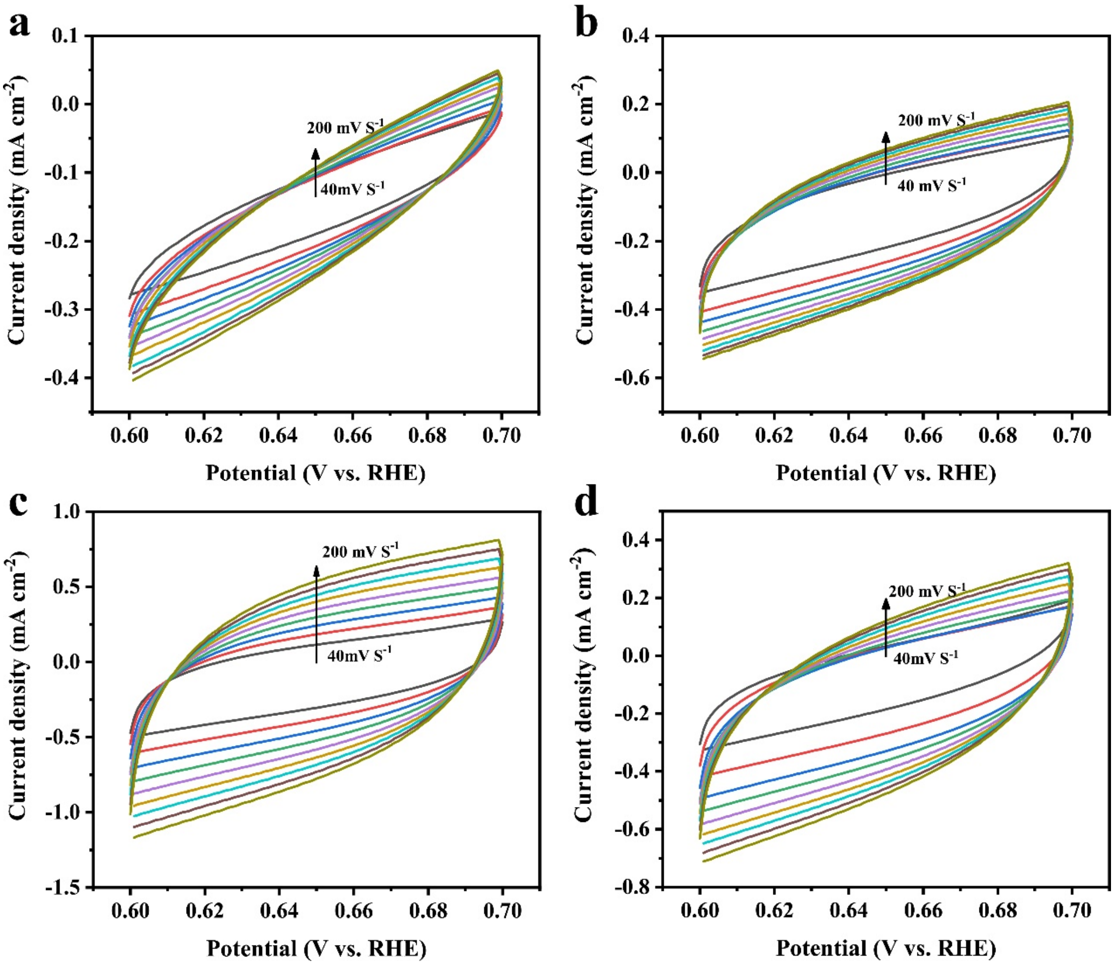


**Figure S6. (a–d)** Cyclic voltammetry curves of the $MoS_2$/rGO/NiS-X (X=0, 3, 5, 7) in 1 M KOH under 0.6–0.7 V vs. RHE.

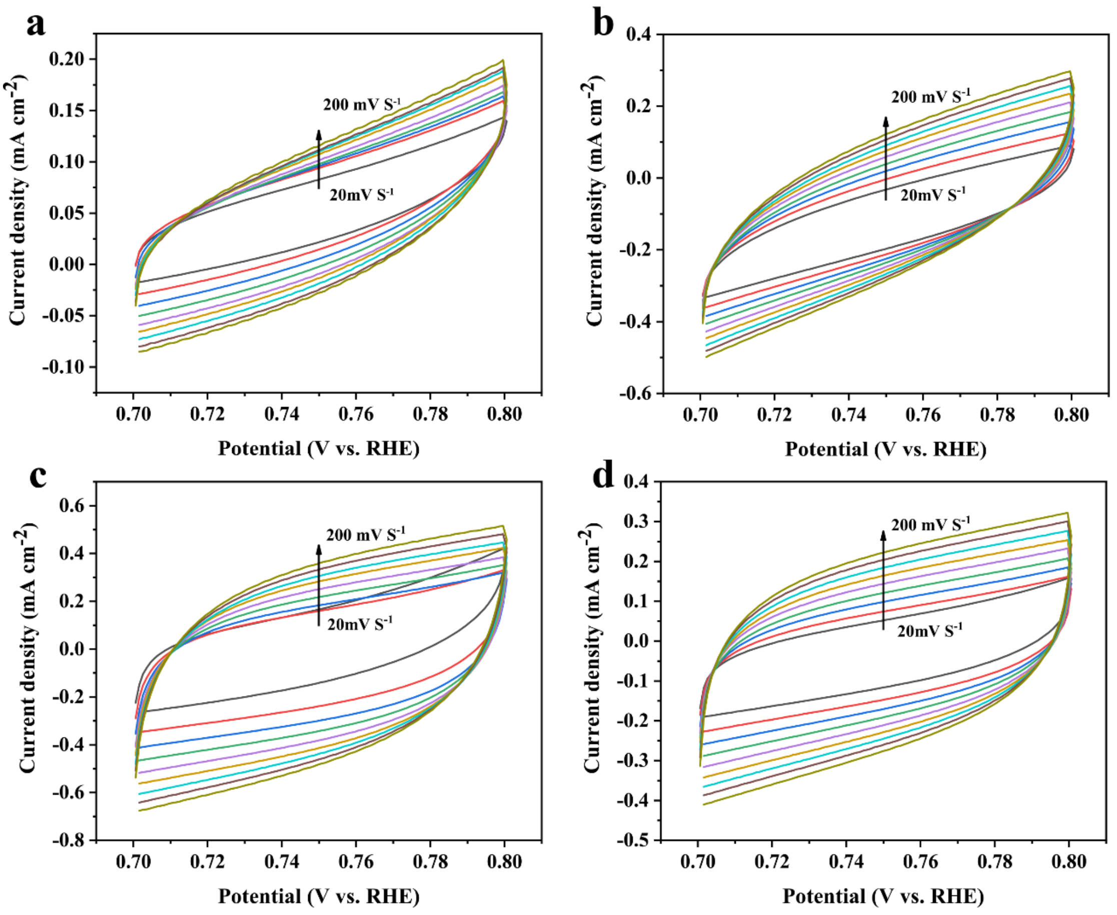


**Figure S7. (a–d)** Cyclic voltammetry curves of the $MoS_2$/rGO/NiS-X (X=0, 3, 5, 7) in 1 M PBS under 0.7–0.8 V vs. RHE.

## 3. Supplementary Tables

**Table S1.** The electrochemical impedance fitting results of as-prepared samples in 0.5 M $H_2SO_4$.

| Sample Name | $R_s$ (ohm) | $R_d$ (ohm) | $R_{ct}$ (ohm) |
|---|---|---|---|
| $MoS_2$/NiS-5 | 19.11 | 31.93 | 515.2 |
| $MoS_2$/rGO/NiS-0 | 13.6 | 21.4 | 93.38 |
| $MoS_2$/rGO/NiS-3 | 13.42 | 26.52 | 145.8 |
| $MoS_2$/rGO/NiS-5 | 15.8 | 4.58 | 19.75 |
| $MoS_2$/rGO/NiS-7 | 16.44 | 31.22 | 111.8 |

**Table S2.** The electrochemical impedance fitting results of as-prepared samples in 1 M KOH.

| Sample name | $R_s$ (ohm) | $R_d$ (ohm) | $R_{ct}$ (ohm) |
|---|---|---|---|
| $MoS_2$/rGO/NiS-0 | 13.82 | 149.6 | 390.6 |
| $MoS_2$/rGO/NiS-3 | 13.63 | 190.4 | 248.9 |
| $MoS_2$/rGO/NiS-5 | 17.4 | 4.58 | 14.03 |
| $MoS_2$/rGO/NiS-7 | 9.747 | 117.6 | 186.4 |

**Table 3.** The electrochemical impedance fitting results of the prepared samples in 1 M PBS.

| Sample Name | $R_s$ (ohm) | $R_d$ (ohm) | $R_{ct}$ (ohm) |
|---|---|---|---|
| $MoS_2$/rGO/NiS-0 | 41.06 | 23.97 | 667.2 |
| $MoS_2$/rGO/NiS-3 | 27.46 | 16.89 | 864 |
| $MoS_2$/rGO/NiS-5 | 32.11 | 3.036 | 105.1 |
| $MoS_2$/rGO/NiS-7 | 24.8 | 49.98 | 532.8 |

## 4. Reference


1. H. Yang, C. Wang, F. Hu, Y. Zhang, H. Lu and Q. Wang, Atomic-scale Pt clusters decorated on porous α-$Ni(OH)_2$ nanowires as highly efficient electrocatalyst for hydrogen evolution reaction. *Science China Materials*, 2017, **60**, 1121-1128.


© 2021 by the authors. Licensee MDPI, Basel, Switzerland. This article is an open access article distributed under the terms and conditions of the Creative Commons Attribution (CC BY) license (http://creativecommons.org/licenses/by/4.0/).
